# Genome-wide DNA copy number profiling and bioinformatics analysis of ovarian cancer reveals key genes and pathways associated with distinct invasive/migratory capabilities

**DOI:** 10.18632/aging.102608

**Published:** 2020-01-02

**Authors:** GuiFen Liu, GuanYu Ruan, MeiMei Huang, LiLi Chen, PengMing Sun

**Affiliations:** 1 Laboratory of Gynaecologic Oncology, Fujian Provincial Maternity and Children’s Hospital, Affiliated Hospital of Fujian Medical University, Fuzhou 350001, Fujian Province, China; 2 Department of Gynaecology, Fujian Provincial Maternity and Children’s Hospital, Affiliated Hospital of Fujian Medical University, Fuzhou 350001, Fujian Province, China

**Keywords:** copy number variations (CNVs), protein/DNA array, ovarian cancer, invasion, metastasis

## Abstract

Ovarian cancer (OC) metastasis presents major hurdles that must be overcome to improve patient outcomes. Recent studies have demonstrated copy number variations (CNVs) frequently contribute to alterations in oncogenic drivers. The present study used a CytoScan HD Array to analyse CNVs and loss of heterozygosity (LOH) in the entire genomes of 6 OC patients and human OC cell lines to determine the genetic target events leading to the distinct invasive/migratory capacities of OC. The results showed that LOH at Xq11.1 and Xp21.1 and gains at 8q21.13 were novel, specific CNVs. Ovarian cancer-related CNVs were then screened by bioinformatics analysis. In addition, transcription factors-target gene interactions were predicted with information from PASTAA analysis. As a result, six genes (i.e., GAB2, AKT1, EGFR, COL6A3, UGT1A1 and UGT1A8) were identified as strong candidates by integrating the above data with gene expression and clinical outcome data. In the transcriptional regulatory network, 4 known cancer-related transcription factors (TFs) interacted with 6 CNV-driven genes. The protein/DNA arrays revealed 3 of these 4 TFs as potential candidate gene-related transcription factors in OC. We then demonstrated that these six genes can serve as potential biomarkers for OC. Further studies are required to elucidate the pathogenesis of OC.

## INTRODUCTION

Ovarian cancer (OC) is a deadly disease that affects women globally. The worldwide incidence of OC is currently 225,500 new diagnoses each year [1–2]. High-grade OC generally grows rapidly, metastasizes early, and has a very aggressive disease course and high rate of chemotherapy resistance [3]. Thus, metastasis and resistance represent major hurdles that must be overcome. Cancer expands clonally and originates from a single clonal state; this expansion is accompanied by genetic changes that lead to functional differences, resulting in different stages and characteristics of tumour development [4–5].

Copy number variations (CNVs) are DNA fragments ranging in size from 1 kilobase (kb) to several megabases (Mb) that arise due to duplication or deletion events. A growing number of studies have reported that CNVs are correlated with the genetic and phenotypic diversity of tumours and are frequently associated with the activation of oncogenic drivers or the deletion of tumour suppressor factors [6–9]. Previous studies using either conventional metaphase chromosome-based comparative genomic hybridization [10–11] or array-based high-resolution genomic technology to identify genome-wide CNVs in OC [12–19] have identified regions of frequently increased copy number along chromosomes 1, 3, 7, 8, 17 and 20 and reduced copy number along chromosomes 1, 4, 13, 16, 18 and X. In addition, many high-level amplifications have been identified as predictive biomarkers, and numerous patient cohort studies have allowed clinicians to accurately characterize genetic changes that predict clinical outcomes [19–20] and precisely compare the genetic alterations between primary and metastatic lesions [15] or histotype-specific OC [21].

In the present study, we carried out an integrated analysis of ovarian cancer using, for the first time, a newly developed, high-resolution genome-wide CNV targeted array to identify CNV-driven genes with bioinformatic tools. The identified CNV-related differentially expressed genes may contribute to the distinct invasive/migratory capacities of OC and serve as potential biomarkers that can improve the accuracy of clinical interpretations and the effectiveness of therapeutics for OC.

## RESULTS

### Comparisons of CNV and LOH frequency between metastatic OC and normal ovarian epithelial tissues

All 4 samples showed chromosomal aberrations. There were, on average, 38.75 chromosomal arm aberrations per specimen among the ovarian tumours and 40.5 aberrations per specimen among the normal tissues (P<0.05). There were ≥40 chromosomal arm aberrations in 100% (20/20) of the normal tissues and 25% (1/4) of the ovarian tumours. There was a similar percentage of LOH between the two groups (Table 1).

**Table 1:** Chromosomal arms affected by CNVs and LOH and the frequencies of CNVs and LOH in tissues.

**Group**	**Sample ID**	**Chromosomal arm**		**Total number**		**Gain**		**Loss**		**LOH**	
		**NO.**	**Average**	**NO.**	**Average**	**NO.**	**Average**	**NO.**	**Average**	**NO.**	**Average**
Cancer	LXM	39	38.75	206	178.5	187	141.75	19	36.75	216	197.25
	WXL	38		103		102		1		198	
	YLH	40		152		147		5		178	
	ZLQ	38		253		131		122		197	
Normal	GRY	41	40.5	167	186.5	42	39.5	125	147	165	155.5
	NDY	40		206		37		169		146	

**Figure 1:**
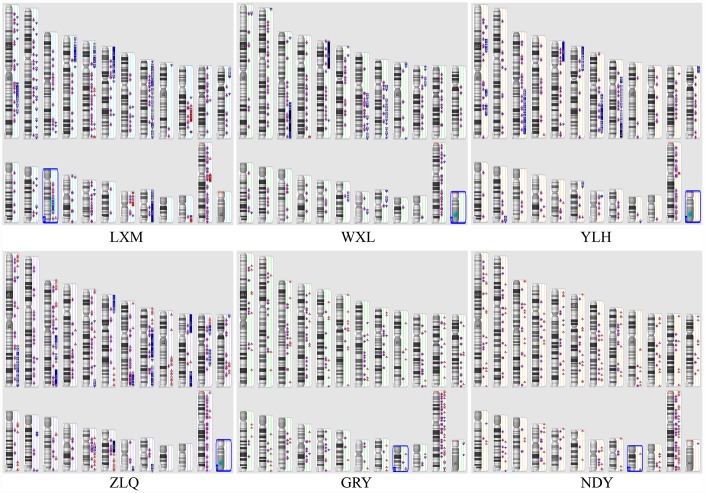
**Illustration of the copy number state of all samples.** Chromosomes 1 through 12 are shown in the upper panel (from left to right), chromosomes 13 through 22 are shown in the lower panel, and chromosomes X and Y are shown in the lower right panel. Six samples were included (LXM, WXL, YLH, and ZLQ: OC; and GRY and NDY: normal epithelial OC). Colour indicates copy number status (blue, duplication; red, deletion; purple, LOH); greater colour saturation indicates greater CNV.

The genome-wide distributions of CNVs are shown in Figure 1. Most of the imbalances in both groups involved LOH. Recurrent, common genomic aberrations between the two groups were observed at several chromosomal intervals. Most notably, CNVs at 7q, 8q, 9q, 12q, 13q, 14q, 16q, 18q, 20q, Xp and Xq were observed in the two groups. The most common CNV regions in the OC samples compared with the normal samples involved both LOH regions, including Xq11.1 and Xp21.1, and amplifications, including 8q21.13, 8q11.21, and 8q23.3. Of these regions, the LOH at Xq11.1 and Xp21.1 and gains at 8q21.13 were novel, specific CNVs.

### Validation of the CNV data in HO-8910 and HO-8910PM cell lines

**Figure 2:**
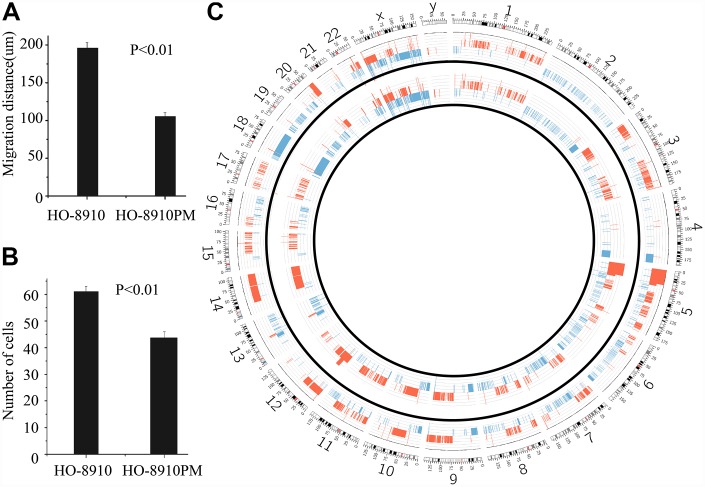
**Different biological behaviours and CNVs in OC cell lines.** The migration distance in the HO-8910 cell line was less than that in the HO-8910PM cell line (p <0.01) (**A**). Cells were incubated on migration wells for 48 h, and the number of cells that migrated to the lower side of the filter was counted (**B**). The results are shown as the mean ± standard deviation (SD) (p <0.01 compared to the HO-8910 cell line). (**C**) Circos plot of the segmented CNVs in HO-8910PM (inner race) and HO-8910 (outer race) cell lines. Coloured bands expanding towards the centre or periphery of the diagram represent copy number losses or gains, respectively (red, gain; blue, loss).

To verify the different biological behaviours of the cellular models *in vitro*, a cell migration assay was performed. The results showed that the migration distances of the HO-8910PM and HO-8910 cell lines at 24 h were significantly different, with distances of 189.22 ± 6.56 μm and 102.31±4.35 μm, respectively (t = 17.91, p <0.01) (Figure 2A). The difference in the number of cells permeating the septum between the HO-8910PM (60.21 ±1.78 cells) and HO-8910 (42.79±2.35 cells) groups was significant (t = 25.95, p < 0.01) (Figure 2B).

In the HO-8910 versus HO-8910PM comparison, although the CNVs of both HO-8910 and HO-8910PM cells overlapped significantly, large regions differed between the two subclones. Segments of gain at 1p36.11, 1p34.3, 1p33, 1p32.1, 1q23.1, 1q32.3, 2q35, 3p26.2, 5p12, 8q21.13, 10p11.1, 10q23.33, 12q23.1, 12q23.3, 12q24.32, 17q12 and 20q12 appeared only in HO-8910PM cells (named “HO-8910PM-specific gain”), whereas segments of loss at 1p36.13, 1p13.3, 1q25.1, 2q22.3, 2q23.1, 2q24.2, 4q12, 5p12, 5q31.3, 6q16.2, 8q22.1, 8q24.11, 10p12.32, 10p12.1, 10p11.23, 10q26.11, 13q21.31, 14q11.2, 14q13.1 and 21q21.1 appeared only in HO-8910PM cells (named “HO-8910PM-specific loss”). The CNV profiles are shown in Figure 2C.

### Screening of differentially expressed genes and enrichment analysis in HO-8910PM cells

**Figure 3:**
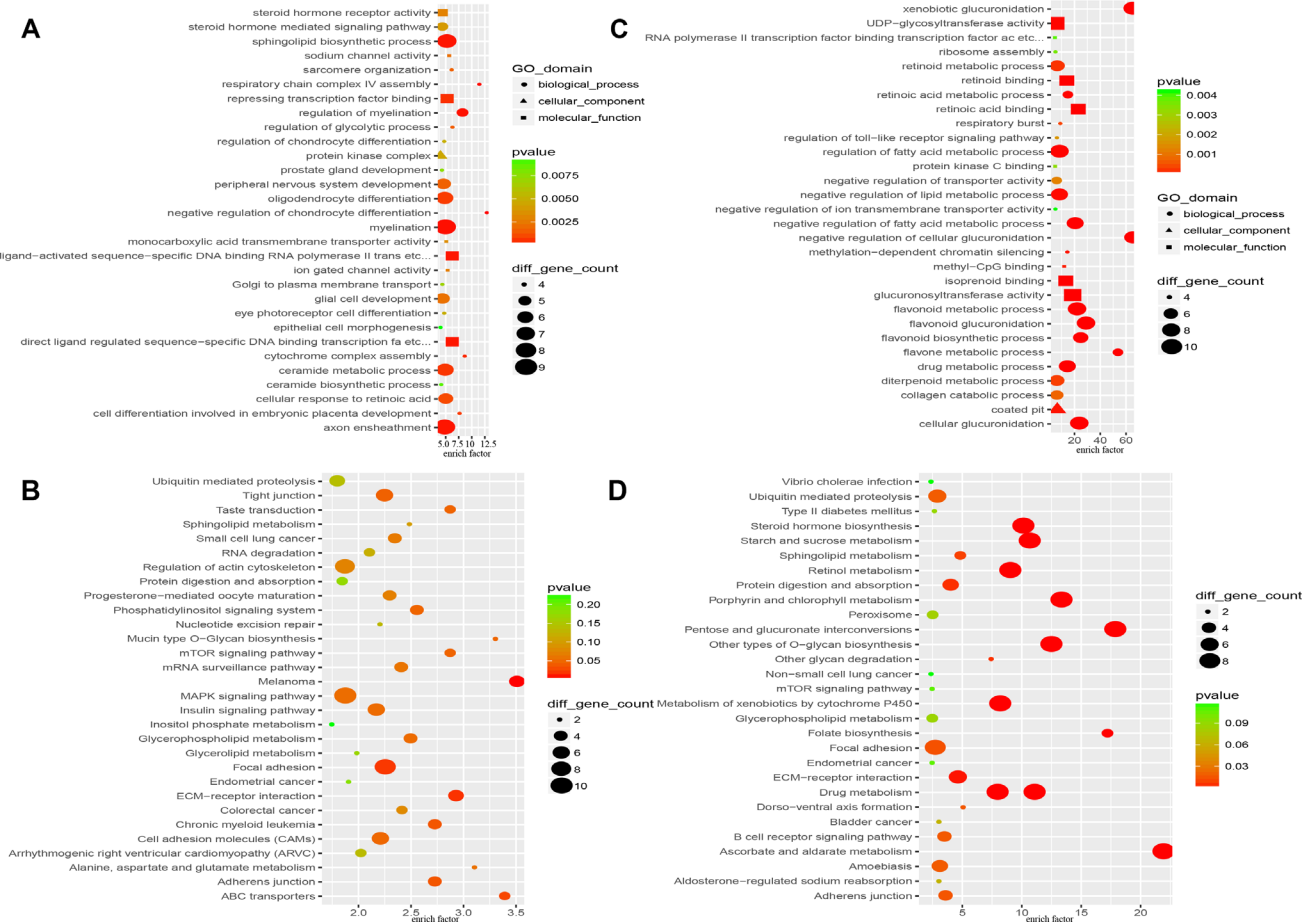
**Functional enrichment analysis of “HO-8910PM-specific gain” and “HO-8910PM-specific loss” genes identified in CNV regions.** (**A**) GO-based annotation was used for the functional enrichment analysis of genes with shared “HO-8910PM-specific gains” (479 genes). (**B**) KEGG pathway analysis of genes with shared “HO-8910PM-specific gains” (479 genes). (**C**) GO-based annotation was used for the functional enrichment analysis of genes with shared “HO-8910PM-specific loss” (400 genes) through DAVID. (**D**) KEGG pathway analysis of genes with shared “HO-8910PM-specific loss” (400 genes). Colour represents the -log of the P value for the significance of enrichment. Only annotations with significant P values < 0.05 are shown.

Functional analysis by the Database for Annotation, Visualization and Integrated Discovery (DAVID) indicated that the proteins encoded by the “HO-8910PM-specific gain” genes (479 genes) are involved in the negative regulation of chondrocyte differentiation, respiratory chain complex IV assembly, myelination, the regulation of myelination, axon ensheathment and other processes (Figure 3A). A total of 479 “HO-8910PM-specific gain” genes were uploaded into the Kyoto Encyclopedia of Genes and Genomes (KEGG) database to conduct KEGG analysis. The significantly enriched KEGG terms were pathways in melanoma, the ECM-receptor interaction, focal adhesion, ABC transporters, adherens junctions, and others (Figure 3B). The proteins encoded by the “HO-8910PM-specific loss” genes (400 genes) displayed significant enrichment for xenobiotic glucuronidation, the negative regulation of cellular glucuronidation, flavonoid glucuronidation, glucuronosyl-transferase activity, cellular glucuronidation, and other terms (Figure 3C). The 400 genes were uploaded for KEGG analysis. The significantly enriched KEGG terms were pathways in ascorbate and aldarate metabolism, pentose and glucuronate interconversions, porphyrin and chlorophyll metabolism, other types of O-glycan biosynthesis, drug metabolism, and other terms (Figure 3D).

### Construction of the protein-protein interaction (PPI) network and cluster identification

**Figure 4:**
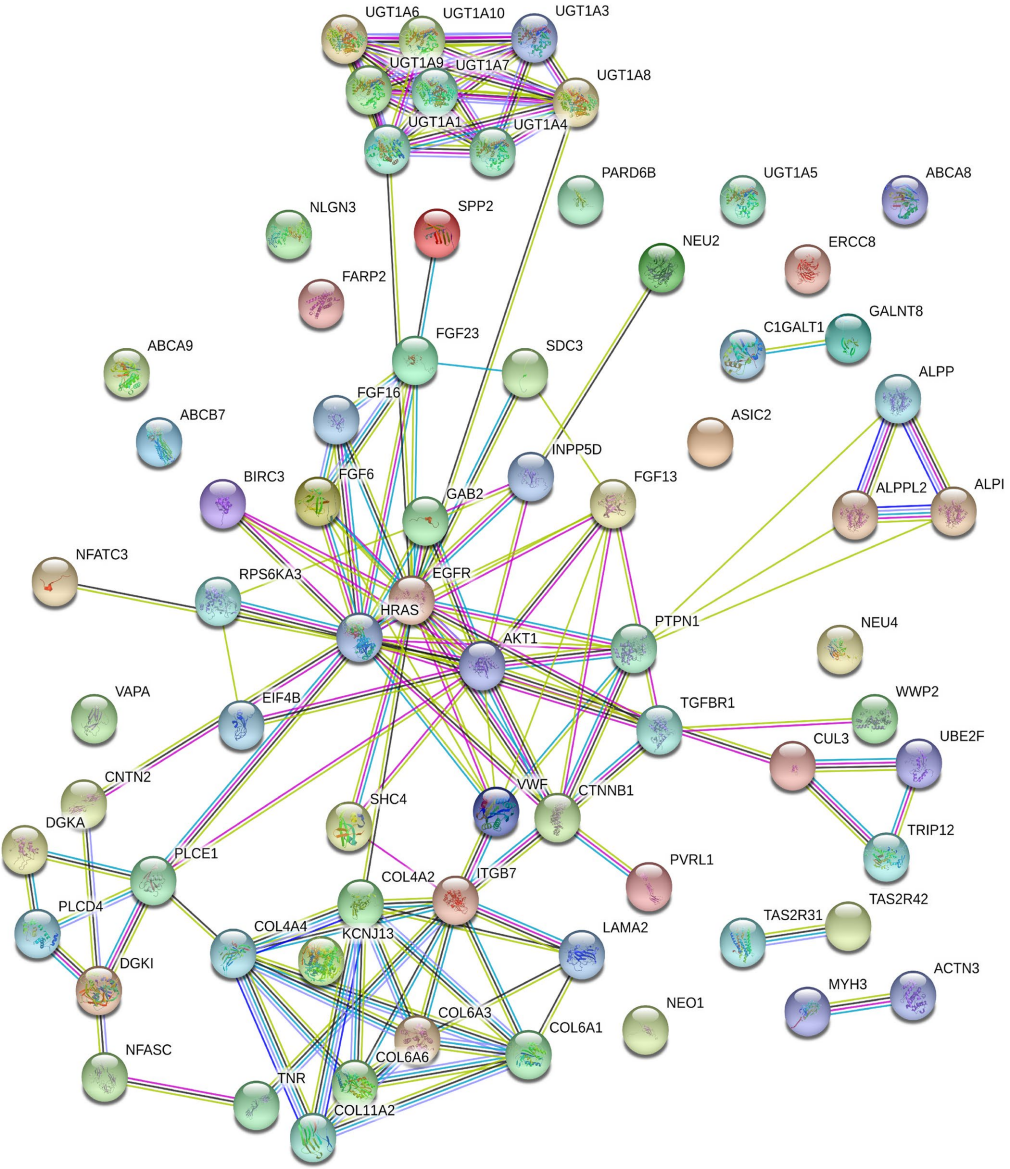
**PPI network based on 71 genes.** There are 54 nodes and 112 edges in the network. Nodes represent genes, and edges represent the interactions between genes.

Among the “HO-8910PM-specific gain” and “HO-8910PM-specific loss” genes enriched in the KEGG database (p>0.05), 71 were shared. To obtain protein interaction data, a human PPI dataset from the Search Tool for the Retrieval of Interacting Genes (STRING) database was applied to the 71 genes. The MCODE algorithm in Cytoscape was used to identify highly interconnected regions or regions of high density in the network. In total, 54 nodes and 112 edges occurred in the PPI network. *GAB2* [22], *AKT1* [23], *EGFR* [24], *CTNNB1* [25]*, HRAS* [26]*, ITGB7* [27]*, COL6A1* [28]*, COL6A3* [29]*, UGT1A1* [30]*, UGT1A10* [31]*, UGT1A3* [32]*, UGT1A4* [33]*, UGT1A6* [34]*, UGT1A7* [35]*, UGT1A8* [36]*,* and *UGT1A9* [37] were the top 16 genes with the highest connectivity degree (Figure 4).

### Assessment of candidate gene expression and patient outcomes

**Figure 5:**
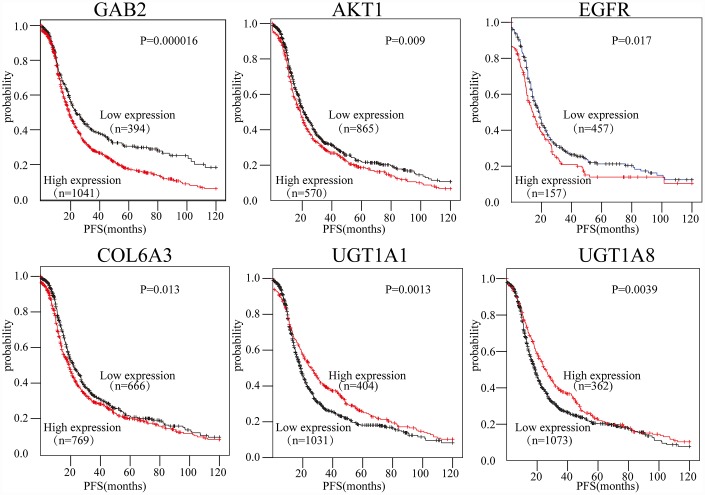
**The expression of six genes predicts the prognosis of OC patients.** The prognostic values of the six genes in OC patients were determined (P < 0.05).

To evaluate the prognostic values of the 16-gene signature selected by the PPI, the Kaplan-Meier plotter (KM plotter) was applied. Progression-free survival (PFS) for patients with OC was determined according to the low and high expression of the hub genes. The results showed that high *GAB2, AKT1, EGFR,* and *COL6A3* expression were associated with poor PFS for OC patients (P<0.05). Additionally, high *UGT1A1* and *UGT1A8* expression were associated with improved PFS for OC patients (P<0.05) (Figure 5). However, the results also showed that the expression levels of *CTNNB1, HRAS, ITGB7, COL6A1, UGT1A10, UGT1A3, UGT1A4, UGT1A6, UGT1A7,* and *UGT1A9* did not correlate with PFS (P>0.05).

### Prognostic significance of *GAB2*, one of the genes of the 16-gene signature in relation to current clinical covariates

To investigate the prognostic significance of the 16-gene signature in association with stage and grade in all validation datasets, we chose *GAB2*, for which the most statistically significant differences had been identified, for univariate and multivariate Cox regression analyses. *GAB2* was a stronger predictor of PFS than grade and stage in both the univariate and multivariate analyses (HR 1.473, 95% CI 1.229-1.766, P <0.01; and HR 1.423, 95% CI 1.989-2.047, p = 0.003 respectively; Table 2).

**Table 2:** Univariate and multivariate Cox proportional hazard regression analyses in all combined validation datasets.

**Variable**	**Univariate analysis**			**Multivariate analysis**		
	**HR**	**95% CI**	**P value**	**HR**	**95% CI**	**P value**
pathology	0.604	0.424-0.860	0.005	0.748	0.618-0.904	0.057
Stage	1.467	1.190-1.806	<0.01	1.347	1.088-1.688	0.006
Grade	0.724	0.545-0.962	0.026	0.795	0.597-1.058	0.115
GAB2	1.473	1.229-1.766	<0.01	1.423	0.989-2.047	0.003

Current clinicopathological factors in ovarian cancer including pathology, stage and grade analyzed in association with the GAB2 gene signature in all combined validation datasets. (HR) hazard ratio; (CI) confdent interval.

### TFs associated with the 6-gene signature

**Figure 6:**
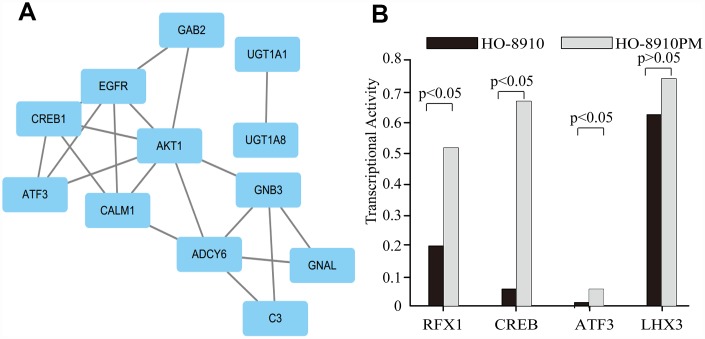
**The target genes and their predicted TFs.** (**A**) The clusters identified in the PPI network containing the target genes and their predicted TFs. (**B**) The transcriptional activities of four TFs.

Four candidate gene-related TFs associated with OC, namely, RFX1, ATF3, CREB, and LHX3, were predicted with a PASTAA analysis (p<0.01). The TF-target gene regulatory network was constructed by introducing the TF and target gene into Cytoscape software (Figure 6A). Relative to the corresponding expression in HO-8910 cells, RFX1, ATF3, and CREB, of a total 345 candidate TFs, were upregulated extensively in HO-8910PM cells, but there was no significant difference in LHX3 expression between the two cell lines (Figure 6B).

## DISCUSSION

Previous studies [38–40] have demonstrated heterogeneity among OCs. The aim of the present study was to compare overall CNVs and LOH across the entire genome using the limiting dilution method [41] to isolate and establish heterogeneous subclones of ovarian cancer tissues and cell lines. The results showed that the most common CNV regions in the OC samples compared with the normal samples were LOH regions, including Xq11.1 and Xp21.1, and amplification regions, including 8q21.13, 8q11.21, and 8q23.3 [42]. Among the CNVs, LOH at Xq11.1 and Xp21.1 and gains at 8q21.13 were novel, specific CNVs. These results suggest that unknown DNA repair genes might be involved in tumour metastasis [43]. However, the HO-8910-derived subclones differed extensively from the normal tissue. Different OC histotypes represented genetic disparities. We hypothesized that heterogeneity may preferentially evolve due to initial alterations in DNA followed by alterations in chromosomal copy number. In the present study, we analysed CNVs in invasive/migratory models. Among the subclones, the HO-8910 and HO-8910PM cell lines, as a pair, serve as *in vitro* research models of OC metastasis. Two cell lines were validated in our previous studies [44–45]. Accordingly, the CNV observed at 8q21.13 between HO-8910PM cells and metastatic OC tissue might be cellular-phenotype related.

CNVs play important roles in the pathogenesis of various types of cancer, such as the CNVs of BRCA, which have been found to be associated with ovarian cancer [46] and breast cancer [47]. In the present study, 16 genes signature [48] with associated copy numbers and differential expression were acquired, of which 6 gene showed the same trends in CNV and were regarded as ovarian cancer-related CNV-driven genes. GAB2 overexpression was associated with poor PFS for OC patients, which was in accordance with previous studies. Overexpression of GAB2 in ovarian cancer cells promotes tumour growth and angiogenesis by upregulating the expression of CXCL1, CXCL2 and CXCL8, which are IKKβ-dependent [49]. Gab2 inhibits E-cadherin expression and enhances the expression of Zeb1, a transcription factor involved in epithelial-to-mesenchymal transition (EMT), and cell migration and invasion through the activation of the PI3K pathway [50]. Co-targeting the IKKβ and PI3K pathways downstream of GAB2 might be a promising therapeutic strategy for ovarian cancer. AKT1 and EGFR play major roles in tumour progression and metastasis in ovarian cancer [51–52]. Davies found that AKT1 (E17K) mutations were effective therapeutic targets for AKT inhibitors [53], although combinations with other targeted agents may be required where activating oncogenic mutations of other proteins were present in the same tumour [54]. Furthermore, alterations in EGFR have been found to perturb enzyme efficacy and proliferation in ovaries and impact susceptibility to OC [55]. COL6A3 was shown to be upregulated in ovarian cancer [56]. Sherman [57] found that the expression of COL6A3 was correlated with cisplatin resistance in ovarian cancer cell lines. Furthermore, highly or moderately differentiated ovarian tumours expressed lower levels of COL6A3 than poorly differentiated tumours, which indicated that the expression of COL6A3 was associated with the grade of the ovarian tumour [57]. There are no reports to date on the relationship between COL6A3 and the metastasis of ovarian cancer. COL6A3 may be a new biomarker for the metastasis of OC. However, UGT1A1 and UGT1A8 over-expression were associated with improved PFS in OC patients. UGT1A1, which has three different variants (UGT1A1*6, UGT1A1*27 and UGT1A1*28), mediates the glucuronidation of SN-38 and CYP3A4, which results in several pharmacologically inactive oxidation products. Homozygosity for UGT1A1*6 or heterozygosity for UGT1A1*6/*28 was associated with a high risk of severe absolute neutrophil count decrease or diarrhoea. In addition to UGT1A1*28, UGT1A1*6 might be a key candidate for determining the dose of combination chemotherapy with irinotecan and cisplatin [58]. The presence of UGT1A1*28 results in an increased risk of ovarian cancer. UGT1A1*28 is associated with mucinous carcinoma and may have a role in the formation of specific histologic sub-groups. It is a potential marker to be considered when planning ovarian cancer chemotherapy [59]. There are no reports to date regarding UGT1A8 in ovarian cancer. There is one study of recombinant human UDP-glucuronosyltransferases (UGTs), including UGT1A1, but Zhou [60] found that UGT1A8 gene polymorphisms can affect the activity of the UDP glucuronosyltransferase enzyme, which may influence the elimination of mycophenolate mofetil in different patients. The 6 CNV-driven genes identified in the present study may be potential markers for ovarian cancer.

In the transcriptional regulatory network, RFX1, ATF3, CREB, and LHX3 were predicted as OC candidate gene-related TFs in the PASTAA analysis. Only RFX1, ATF3, and CREB showed two-fold increases in expression in HO-8910PM/HO-8910 cells, which was validated with protein/DNA arrays. Consistent with these results, Subbiah reported that CREB, an important TF for neurotrophin activity, employed signalling pathways mediated by PI3-kinase and p38 MAPK, whereas Akt, the downstream component of the PI3-kinase pathway, is known to regulate the covalent modification of cytosolic proteins, such as glycogen synthase and the proapoptotic protein BAD, in pheochromocytoma (PC12) cells [61]. The phosphoinositide 3-kinase protein kinase B (PI3K-Akt) signalling pathway was involved in the formation and progression of many malignancies due to genetic alterations in their components or the activation of upstream cell surface receptors [62]. Future studies are needed to explore the implications of each of signalling pathway on CREB-responsive genes important for cell cycle regulation, survival, and differentiation in OC. It has been observed that COL6A3 acts as an oncogene in cancer and that the antagonism of COL6A3 could be developed as an effective therapeutic treatment for gastric cancer [63]. The number of cells that expressed COL6A3 relative to normal cells, Akt and PI3K were markedly decreased. COL6A3 gene silencing inhibits gastric cancer cell proliferation, migration, and invasion and promotes apoptosis through the PI3K-Akt signalling pathways [64]. The present need to be confirmed before the clinical application of COL6A3 and the CREB and PI3K-Akt signalling pathways in patients with OC. However, the association between other hub genes and OC has not been widely reported and needs further investigation.

In summary, we systematically analysed the functions of altered genes in OC cell line models with distinct invasive/migratory capacities. Data on copy number diversity were integrated with clinical outcomes and DNA/protein expression to facilitate the search for potential therapeutic targets. We hypothesize that clone-specific functional and genetic profiling will be helpful methods for identifying new molecular pathways underlying cancer and new biomarkers for clinical applications. This information will not only reveal the heterogeneity of OC at different developmental stages but also elucidate other factors that contribute to cancer-related deaths, such as genes that promote invasion/migration.

## MATERIALS AND METHODS

### Patients

The samples analysed in this study were tissue bank samples from 4 OC patients with lymph node metastasis and samples of normal ovarian surface epithelia from 2 non-OC patients the Fujian Maternity and Children Health Hospital (Fujian, China). Tissues were collected after the time of surgery. Four patients with metastatic OC who received surgical treatment between May 2013 and January 2015 were selected. Metastatic OC was identified intraoperatively and postoperatively. The patients’ ages ranged between 55 and 60 years. The postoperative pathology revealed 2 serous adenocarcinomas, a mucinous adenocarcinoma, and a serous cystadenocarcinoma. Normal ovarian epithelial tissue was obtained from two patients that had undergone bilateral appendage removal due to other benign gynaecological diseases. The ages of these 2 patients were 39 years and 47 years. All participants provided written informed consent, and all study protocols were approved by the Ethics Committee of the Fujian Maternity and Children Health Hospital (Fujian, China) (approval number 2013004). All methods of data collection and analysis were performed in accordance with relevant guidelines and regulations and with appropriate quality control.

### CNV assay

Tumour samples were obtained from each patient, and DNA was extracted using a QIAamp DNA Blood Midi Kit (Qiagen, Duesseldorf, Germany) following the manufacturer’s instructions. The CNVs were detected with a CytoScan HD microarray platform (Affymetrix, Santa Clara, CA, USA) and a high-density chip that contained 2,636,550 probes. All samples passed initial quality control. The array data were analysed using the Chromosome Analysis Suite (ChAS) software package [65], and annotations were performed with the Genome Reference Consortium (GRC) human reference genome version GRCh37 (hg19). The data were filtered such that only those regions larger than 50 kb comprising at least 25 contiguous markers were retained.

### Data analysis

To calculate the CNV, the data were normalized to baseline reference intensities using 270 HapMap samples and data from 90 healthy normal individuals included in the software. The Hidden Markov Model (HMM) (available within the software package) was used to determine the copy number states and their breakpoints. The thresholds of log2 ratio ≥1.5 and ≤-1.5 (as suggested by the software) were used to categorize the altered regions as CNV gains (amplifications) and copy number losses (deletions), respectively.

To prevent the detection of false-positive CNVs arising due to inherent microarray “noise”, only alterations that involved at least 25 consecutive probes and that were >50 kb in length were considered in the analysis of gains or losses in our study. Amplifications and deletions were analysed separately. The detected CNVs were evaluated separately in terms of frequency and length. To exclude aberrations representing common, normal CNVs, all the identified CNVs were compared with those reported in the Database of Genomic Variants (DGV, http://projects.tcag.ca/variation/).

To identify genes involved in CNVs, the UCSC (http://genome.ucsc.edu), Ensemble (http://www.ensembl.org), and BioGPS databases (http://biogps.org) were queried. The gene annotations and overlaps were determined using the human genome build 19 (hg19) and several widely used online databases (Ensembl: http://www.ensembl.org; UCSC: http://genome.ucsc.edu; and NetAffx: http://www.affymetrix.com).

For the LOH analysis, the LOH algorithm in the genotyping console 2.0 was used. Regions of LOH/copy number LOH (cnLOH) that were >3 Mb were identified and analysed further.

### Pathway and functional enrichment analyses

To investigate the biological characteristics of the overlapping genes in the detected CNV regions, GO and KEGG pathway enrichment analyses were performed using DAVID [66, 67]. The cut off criterion was set as P < 0.05.

### Cell lines and cell cultures

The OC cell line HO-8910 and its metastatic equivalent, HO-8910PM [68], were purchased from the Type Culture Collection Center of the Chinese Academy of Science (Shanghai, China). All cell lines were cultured in Dulbecco’s modified Eagle’s medium (DMEM) (Gibco; Thermo Fisher Scientific, Inc., Waltham, MA, United States) supplemented with 10% foetal bovine serum (Gibco), 1% penicillin and 1% streptomycin (100 IU/ml) in a 37°C incubator with 5% CO_2_.

### Cell scratch assay

The horizontal migration of cells was assessed with a scratch assay performed according to a previous report [69] with slight modifications. Cells were seeded at a density of 5.0 × 10^5^ cells/well and imaged at 40× magnification with an Olympus IX70 inverted fluorescence microscope (Olympus Corporation, Japan) at 0 and 24 h post-scratching. Image-Pro Express C software 5.1 (Olympus Corporation) was used to measure the change in cell distance between scratches. The average horizontal migration rate was calculated using the following formula: (width at 0 h - width at 24 h) / width at 0 h × 100.

### Transwell chamber assay

The invasive capacity of the cell lines was determined using a Matrigel invasion chamber assay. Cells were seeded at a density of 5.0×10^5^ cells/well. The number of cells on the underside of the filter was determined by counting cells in five random fields from three filters for each treatment at 200× magnification with an inverted microscope (Olympus Corporation).

### PPI network construction and cluster identification

PPI networks may represent molecular complexes. The PPI network of the genes was constructed using the STRING database (https://string-db.org). Upon entering a single protein name, multiple names or an amino acid sequence in the STRING website, the STRING resource can construct a network of protein interactions. The network view summarizes the network of the predicted associations for a particular group of proteins. The network nodes represent proteins, and edges represent the predicted functional associations. Additionally, in the legend section, a list of inputs is shown, and the predicted associations are shown in a list below the input, sorted by score. Subsequently, the results can be visualized using Cytoscape software. The cutoff criterion of the confidence score was set as > 0.7. In addition, the molecular complex in the global PPI network was obtained with MCODE. The screening options were set as follows: degree cutoff = 2, node score cutoff =0.2, k-core = 2, and max. depth= 100.

### Survival analysis

The KM plotter is a web tool that predicts the effect of genes on survival (http://kmplot.com/analysis/index.php?p=background). After entering the genes of interest into the website, we divided the patients into two groups according to the expression level of each gene and statistically analysed survival rate. Univariate and multivariate Cox proportional hazard regression analyses were performed to evaluate independent prognostic factors associated with survival. The gene signature, pathology, tumour grade, and FIGO stage were employed as covariates. The hazard ratio (HR) with 95% CIs and the log-rank P value were calculated and shown.

### Prediction of TFs

The relevant TFs of the differentially expressed genes were predicted based on the PASTAA analysis [70]. The p value calculated by the hypergeometric test was used to assess the significance of the overlap between each OC gene and the predicted target of the TF. Next, a TRAP analysis was performed to predict the affinity relationship between the gene and the relevant TF. The TFs and the differentially expressed genes were introduced into Cytoscape software to construct a gene-TF coexpression network.

### Nuclear and cytoplasmic protein extraction

Nuclear and cytoplasmic proteins were extracted from adherent cells at 70–90% confluence according the instructions provided with the NuCLEARTM Extraction Kit (Sigma, United States). In summary, the cells were rinsed with fresh phosphate-buffered saline (PBS), the appropriate amounts of reagents were added to the particular packed-cell volume, the cells were lysed, and the cytoplasmic and nuclear proteins were isolated by gradient centrifugation. Protein concentrations were quantitated with a BCATM assay kit from Pierce (United States).

### Protein/DNA arrays

HO-8910 and HO-8910PM cell cultures were screened for TFs using the TranSignal Protein/DNA Array (Panomics, United States) following the manufacturer’s protocol. Briefly, proteins were isolated as described above, and nuclear extracts (25 μg) were incubated with the provided biotin-DNA probe mix for 30 minutes. Protein-DNA complexes were isolated with 2% agarose gel electrophoresis. The proteins were eliminated from the complex, and the biotin-DNA was hybridized to the membranes containing the consensus binding sequences of the TFs. Next, the membranes were incubated with streptavidin-alkaline phosphatase conjugate. Signals of the hybridized probes were visualized using the chemiluminescent imaging system provided with the TranSignal Protein/DNA Array Kit and exposed to X-ray film [71]. Using the expression in HO-8910 cells as the benchmark, a two-fold increase or decrease in expression in HO-8910PM/HO-8910 cells was considered significant. Experiments were performed in duplicate.

### Statistical analysis

Statistical analysis of the data was performed with SPSS 17.0 software (SPSS, Chicago, Illinois, USA). The data are expressed as the means ± standard errors of the mean (SEM). The significance of differences in values was evaluated through analysis of variance (ANOVA) or an unpaired two-tailed Student’s t-test. A P value < 0.05 was considered to indicate a significant difference. All experiments were repeated at least three times.
